# Translation and validation of the Chinese version of the Self-awareness Scale for Nurses

**DOI:** 10.3389/fpubh.2024.1352983

**Published:** 2024-04-17

**Authors:** Qing Chen, Chunguang Liang, Jing Lu, Zhaoquan Jiang

**Affiliations:** ^1^Department of Nursing, Jinzhou Medical University, Jinzhou, China; ^2^The Second Hospital of Chaoyang, Liaoning, China; ^3^Department of Nursing, Jinzhou Medical University, Jinzhou, China

**Keywords:** self-awareness, nurse, scale, reliability, validity

## Abstract

**Background:**

Levels of self-awareness may affect the decision-making ability of clinical nurses and may also be related to mental health. Therefore, it is crucial to develop tools to identify nurses’ level of self-awareness. The purpose of this study was to investigate the reliability and validity of a short scale among Chinese nurses and to explore the factors associated with nurses’ self-awareness.

**Methods:**

A total of 957 participants were recruited, 549 participants were used for reliability tests and 408 subjects were used for impact factor studies. They completed the General Information Questionnaire, the Self-Awareness Scale for Nurses, and the Psychological Distress Scale. Exploratory factor analysis, confirmatory factor analysis, Cronbach’s alpha, and retest reliability were used to investigate the psychometric properties of the Self-Awareness Scale for Nurses. Multiple regression analyses were used in this study to investigate the relationship between nurses’ self-awareness and the independent variables.

**Results:**

A 4-factor model of the Chinese version of the Self-Awareness Scale for Nurses was validated. The overall Cronbach’s alpha value for the Chinese version of the Self-Awareness Scale for Nurses was 0.873. Cronbach’s alpha values for each subscale ranged from 0.808 to 0.979. Significant predictors of each dimension of the Self-awareness and the total score of the scale were age and work experience.

**Conclusion:**

The Chinese version of the Self-Awareness Scale for Nurses is a valid and reliable scale.

## 1 Introduction

Self-awareness constitutes a fundamental difference between humans and other living species (1). Self-awareness is often regarded as a distinguishing characteristic of human beings and it can be explained as this state in which a person can be aware of his or her own thinking and behavior (2). Self-awareness begins in infancy and continues into adulthood, and it develops through personal evaluation and reflection, and recollection of past events (3). Having self-awareness can help individuals better manage their business, work, and daily lives (4). Through a review of the relevant literature, we found that self-awareness among nurses is particularly critical.

Self-awareness is of extremely high importance for nurses, due to the fact that certain nursing associations view self-awareness as a core indicator of nursing competence (5, 6). Self-awareness is not only a key competency in the nurse–patient relationship, but it also forms a foundational trait for nurses (7, 8). References state that self-awareness is an evolving process that involves the development of nurses in clinical thinking and decision-making skills (9, 10). However, a lack of self-awareness among nurses may hinder the development of a strong doctor-patient relationship, which ultimately affects the quality of care provided to patients (11). A recent descriptive study (12) highlighted the need for nurses to focus on self-awareness and the importance of how to appropriately respond to the various challenges that may arise. Self-awareness is of greater importance in assessing the nursing competence of caregivers, and it can also have a direct impact on patient prognosis.

Nowadays, measurement tools related to self-awareness are available both nationally and internationally. For example, American scholars Fenigstein et al. (13) developed and validated the Self-Consciousness Scale (SCS) in 1975, which includes public and private self-awareness. Later, the scale was widely used. In the absence of stable and efficient tools, Smeets and his team (14) point out that experts in the health professions can use them to assess various types (or levels) of self-awareness in various areas or domains of daily life. Thus Winkens et al. (15) developed and validated the Self-Awareness of Daily Life Scale (SADL-3), which was developed to assess self-awareness in the chronic period after acquired brain injury (ABI), including family relationships, friends and social interactions, intimacy and sexuality, leisure time, work and daytime activities, housing situation and living conditions, and health and appearance seven domains of daily living to assess patients. There is also the Emotional Self-awareness Questionnaire (ESQ) (16) developed by kill et al. which measures emotional intelligence and consists of 11 subscales: mood, self-reflection, empathy, emotion management, adaptability, motivation, self-esteem, self-efficacy, interpersonal relationships, affect and counseling. And the Self-Awareness Outcome Questionnaire (SAOQ) (17), which measures the impact of self-awareness on daily life and consists of four subscales: reflective self-development, acceptance of self and others, work initiative, and the emotional cost of self-awareness. In an academic editorial, Kuwano and McMaster (18) emphasized the need for more research to explore and assess self-awareness in care settings.

Rasheed and his team (19) first designed and validated an instrument called the Self-Awareness Scale for Nurses (SASN) in 2020, which combines qualitative and quantitative research methods and consists of 18 different items and four different dimensions. Nonetheless, this instrument has not yet been introduced to China, so more in-depth research is needed to confirm its applicability in this new cultural context. The aim of this study was to translate the original Self-Awareness Scale for Nurses (SASN) into a Chinese version and to validate the SASN.

## 2 Methods

### 2.1 Study design and participants

From April 2022 to August 2022, a cross-sectional study was conducted to validate the Self-Awareness Scale for Nurses. It consisted of two phases: (1) psychometric assessment of the scale; (2) analysis of factors influencing nurses’ self-awareness.

In April 2022, the method of cross-sectional study was used. Nurses from four hospitals in Liaoning Province participated in the study. Inclusion criteria: Registered nurses working in healthcare facilities, volunteered for the study. Intern nurses were excluded.

The number of nurses required is based on 10 times the number of items, the scale has 18 items and the sample size should be at least 90–180 (20). The survey was anonymous, but we flagged 30 nurses who voluntarily participated in a second experiment for test–retest reliability 2 weeks later.

### 2.2 Data collection

The survey took place between April and June 2022. We invited the head nurses from each hospital to assist with the investigation. We first sent the questionnaire to the corresponding head nurses, and then they sent the questionnaire to the nurses in the department. In this study, participants were asked to fill in questionnaires according to the actual situation. Filling out the questionnaire is voluntary. A total of 554 nurses participated in this study and finally, 549 questionnaires were retained after data compilation.

In addition, to explore the influencing factors of nurses’ self-awareness, collected another 430 data from six hospitals from July to August 2022.

### 2.3 Instruments

The study questionnaire consisted of demographic information and original scale, and Kessler 10 psychological distress scale (K10). The demographic information included age, gender, marital status, education levels, average monthly income (yuan), working experience (years), and job title.

The 18-SASN was developed by Rasheed et al. (19) and consists of four subscales: contextual, conscientious, personal, and professional awareness. Each item corresponds to a response ranging from 5 (always) to 0 (never). The overall Cronbach’s alpha coefficient for the original scale was 0.87 and content validity was 0.94 (19).

The Kessler 10 psychological distress scale (K10), used by the World Health Organization in several countries around the world, assesses the level of non-specific psychological distress by asking about the frequency of non-specific psychological symptoms in the past 4 weeks. The K10 was first applied to China by Xu et al. (21), this scale consists of 10 items, including subscales measuring anxiety and depression. The internal consistency coefficient for this scale was 0.80. Each item was rated using a scale of 1 (hardly ever) to 5 (all the time). The total score is divided into four levels, the higher the level, the higher the risk of psychological disorders: 10–19 (low), 20–24 (low-moderate), 25–29 (moderate), and 30–50 (high).

### 2.4 Translation process

Following the consent of the original author, we conducted our translation process using the Brislin (22) principle, which involves ensuring the accuracy and cultural relevance of the translation without altering the meaning. First, it was translated into Chinese by two experts in English, forming two separate versions (A1 and A2). Next, the two experts and researchers compared the Chinese translations of A1 and A2, discussed and corrected the inconsistencies, and obtained the first draft of the Chinese version (A12). Then, we invited two nursing experts who had no contact with SASN to back-translate the Chinese draft into English to form back-translated versions (B1 and B2). Finally, the experts compared and discussed the original scale, the first draft of the Chinese translation, and the back-translated English scale. Based on the experience, Chinese expression habits, and customary concepts, a tentative draft of the Chinese version was formed (T1).

A pre-test was conducted with 30 nurses, and the final Chinese version (T2) was obtained through a collaborative discussion between team members and translation experts.

### 2.5 Statistical analysis

Data were statistically analyzed using SPSS 25.0 and Amos 24.0 software. The reliability and validity of 18 items were tested. Quantitative data were represented by (mean, standard deviation), and classified data were represented by frequency and component ratio. Validity evaluation indicators include exploratory factor analysis (EFA) and confirmatory factor analysis (CFA), content validity index (I-CVI), and the average value of all content validity indicators of the scale (S-CVI/Ave), and discriminant validity. Reliability was tested using Cronbach α coefficient and test–retest reliability coefficient. To investigate potential independent variables of the nurses’ self-awareness, a multivariate linear regression analysis was utilized. For all analyses, *p* < 0.05 was considered statistically significant.

#### 2.5.1 Item analysis

The total score of SASN was sorted from high to low, and the respective top 27% and bottom 27% were defined as high and low groups. Items in both groups were analyzed using independent samples *t*-test, and if the scores for each item in both groups were statistically significant (*p* < 0.05), the items were deemed to have better discriminatory power.

Correlation of items with total scores, correlation of corrected items with total scores, and Cronbach’s coefficient of the scale after removal of items.

#### 2.5.2 Content validity

The content validity evaluation indexes included the content validity index (I-CVI) and the mean value of all content validity indexes of the scale (S-CVI/Ave). I-CVI greater than 0.78 and S-CVI/Ave greater than 0.90 indicate an acceptable range (23, 24).

#### 2.5.3 Construct validity

Factor analysis consists of two aspects: exploratory factor analysis (EFA) and confirmatory factor analysis (CFA). The total sample was randomly divided into two parts, with EFA using the first data group (*n* = 261) and CFA using the second data group (*n* = 288). Exploratory factor analysis was mainly used for screening items and dimension division. Performed the Bartlett test (25) of sphericity on all items and calculated the Kaiser-Meyer-Olkin (KMO) index (26). The Bartlett test for sphericity was significant (*p* < 0.05), and the KMO >0.7 was considered suitable for factor analysis. Factor rotation was performed using the variance maximization method of rotated principal component analysis (PCA). In general, factor retention criteria were based on the following: (1) factor eigenvalue >1; (2) factor loading >0.4; (3) each factor is at least contained three items (25, 26).

Based on the results of the exploratory factor analysis, a confirmatory factor analysis (*n* = 288) was performed on the factor model. The following metrics were used to assess the fit of the structural model: squared degrees of freedom (*χ*^2^/df), the root mean square error of approximation (RMSEA), the goodness of fit index (GFI), the standardized root mean square residuals (SRMR), the comparative fit index (CFI), and the Tucker Lewis Index (TLI). Fitting models should have the following characteristics: *χ*^2^/df < 3, RMSEA and SRMR <0.08, GFI, CFI, and TLI > 0.9 (27, 28).

#### 2.5.4 Reliability analysis

The internal consistency reliability of the assessment scale was determined by the Cronbach alpha coefficient, the correction term-total correlation, and the test–retest reliability. The acceptable value of the Cronbach alpha coefficient is equal to or greater than 0.70 (29). The test–retest reliability of the scale was reflected by calculating the intraclass correlation coefficient (ICC). After 2 weeks, the 30 nurses who had been flagged in the prior trial were retested to determine the reproducibility of the results. Spearman correlations were used to analyze the correlations, with a correlation coefficient larger than 0.7 serving as the criterion (29).

#### 2.5.5 Analysis of influencing factors

The independent variables age, gender, education level, average monthly income, work experience, job title, and psychological distress scores were included in this study, and the analysis method of multiple linear regression was used to explore the factors influencing nurses’ self-awareness.

## 3 Ethics statement

In this study, all procedures were carried out in accordance with the 1964 Declaration of Helsinki, and the study protocol was approved by the Ethics Committee of Jinzhou Medical University (approval number: JZMULL2021009). Informed consent was obtained from all participants.

## 4 Results

### 4.1 Descriptive statistical

The demographic characteristics of the participants in the cultural adaptation phase of the study are shown in Supplementary materials. In the research phase of the influencing factors analysis, the majority of the nurses were female (91.7%), 48.5% had more than 11 years of work experience, 48.3% of the nurses had the title of Charge Nurse and above, and the other information is shown in Table 1.

**Table 1:** Demographic characteristics (*N* = 408).

Variables		*n* (%)
Gender	Male	34 (8.3)
	Female	374 (91.7)
Age ( $\bar{x}$ ±s)		33.77 ± 6.49
Education level	Technical secondary school	6 (1.5)
	Junior college	67 (16.4)
	Undergraduate	327 (80.1)
	Postgraduate and above	8 (2.0)
Average monthly salary (yuan)	<3,000	25 (6.1)
	3,000- <5,000	221 (54.2)
	5,000- <10,000	143 (35.0)
	≥10,000	19 (4.7)
Working experience (year)	1–5	113 (27.7)
	6–10	97 (23.8)
	≥11	198 (48.5)
Job title	Nurse	76 (18.6)
	Nurse practitioner	135 (33.7)
	Nurse practitioner in charge and above	197 (48.3)
Psychological distress (K10) score ( $\bar{x}$ ±s)		26.20 ± 10.63

### 4.2 Item analysis

Analyzed all items in the SASN (18 items). In this study, the top 27% of the high subgroup and the bottom 27% of the low subgroup cutoff values were 64.0 and 76.0. The results of the independent samples t-test showed statistically significant scores for each item in both groups (*p* < 0.05) (Table 2).

**Table 2:** Comparison of scores between high and low scores (*N* = 309).

Items	High-score Group (*n* = 158), mean (SD)	Low-score group (*n* = 151), mean (SD)	*t*-test (df)	*p*-value
V1	4.35 (0.881)	2.97 (0.770)	14.695 (304.571)	<0.001
V2	4.19 (0.965)	2.95 (0.715)	12.831 (289.221)	<0.001
V3	4.02 (0.913)	2.93 (0.780)	11.246 (303.260)	<0.001
V4	4.61 (0.756)	3.18 (1.144)	12.896 (258.345)	<0.001
V5	5.00 (0.000)	4.53 (0.764)	7.560 (150.000)	<0.001
V6	4.99 (0.080)	4.40 (0.818)	8.819 (152.713)	<0.001
V7	4.99 (0.080)	4.54 (0.710)	7.870 (153.603)	<0.001
V8	4.95 (0.247)	3.89 (1.074)	11.852 (165.165)	<0.001
V9	4.89 (0.356)	3.64 (1.036)	14.057 (183.623)	<0.001
V10	4.88 (0.345)	3.65 (1.078)	13.385 (179.192)	<0.001
V11	4.19 (1.253)	3.60 (1.120)	4.337 (307.000)	<0.001
V12	4.68 (0.609)	3.71 (0.914)	10.988 (259.647)	<0.001
V13	4.75 (0.436)	2.97 (0.816)	23.666 (226.936)	<0.001
V14	4.77 (0.421)	2.99 (0.848)	23.268 (217.336)	<0.001
V15	4.76 (0.429)	3.03 (0.864)	22.182 (217.450)	<0.001
V16	4.74 (0.454)	2.96 (0.791)	24.128 (236.956)	<0.001
V17	4.66 (0.550)	2.90 (0.772)	22.943 (270.085)	<0.001
V18	4.72 (0.464)	2.97 (0.791)	23.558 (239.920)	<0.001

The item-total correlation coefficient after item correction was 0.250 except for item 11, and the remaining items were greater than a criterion of 0.3 (*r* = 0.400–0.622) (29, 30). Also conducted a correlation analysis between the items and the total score of the scale, and the results showed that, except for item 11, the remaining items were significantly and positively correlated with the total score and the correlation was high (*r* = 0.469–0.712, *p* < 0.001). Meanwhile, initially reliability analysis showed that the overall Cronbach *α* coefficient was 0.867 (95% CI: 0.851–0.883), by deleting item 11, the internal consistency of the scale can be improved to 0.873. Item 11 was therefore deleted after consideration by the experts. The results are shown in Table 3.

**Table 3:** Item analysis for Chinese version of the SASN (*N* = 549).

Items	Mean (SD)	Corrected item total correlation	*r*	Cronbach alpha if the item was deleted
V1	3.64 (1.056)	0.446	0.528	0.862
V2	3.57 (1.034)	0.432	0.503	0.863
V3	3.45 (0.982)	0.400	0.470	0.864
V4	3.94 (1.166)	0.413	0.499	0.865
V5	4.84 (0.486)	0.430	0.469	0.864
V6	4.70 (0.550)	0.457	0.501	0.863
V7	4.84 (0.455)	0.452	0.484	0.864
V8	4.47 (0.903)	0.454	0.525	0.862
V9	4.35 (0.907)	0.493	0.562	0.860
V10	4.36 (0.920)	0.557	0.603	0.858
V11	3.93 (1.217)	0.250	0.250	0.873
V12	4.29 (0.896)	0.448	0.495	0.862
V13	3.86 (0.994)	0.620	0.712	0.855
V14	3.88 (1.006)	0.614	0.707	0.855
V15	3.91 (1.008)	0.595	0.692	0.856
V16	3.83 (0.993)	0.622	0.711	0.855
V17	3.81 (1.006)	0.595	0.692	0.856
V18	3.84 (0.988)	0.599	0.696	0.856

### 4.3 Content validity

The results of the content validity analysis showed that the I-CVI for item 11 was 0.571 (Table 4), indicating that item 11 was indeed inappropriate for Chinese nurses. The I-CVI values for the remaining items were from 0.857 to 1.000, and the S-CVI/Ave for the scale was 0.921.

**Table 4:** Content validity analysis for Chinese version of the SASN (*N* = 549).

Item	Experts (score)							I-CVI
	1	2	3	4	5	6	7	
V1	1	1	1	1	1	0	1	0.857
V2	1	1	1	1	1	1	1	1.000
V3	1	1	1	1	0	1	1	0.857
V4	1	1	1	1	1	1	1	1
V5	1	1	1	1	1	1	1	1
V6	1	0	1	1	1	1	1	0.857
V7	1	1	1	1	1	0	1	0.857
V8	1	1	0	1	1	1	1	0.857
V9	1	1	1	1	1	1	1	1
V10	1	1	1	1	1	1	1	1
V11	0	1	0	1	0	1	1	0.571
V12	1	1	0	1	1	1	1	0.857
V13	1	1	1	1	1	1	1	1
V14	1	1	1	1	1	1	1	1
V15	1	1	1	1	0	1	1	0.857
V16	1	1	1	1	1	1	1	1
V17	1	1	1	1	1	1	1	1
V18	1	1	1	1	1	1	1	1

### 4.4 Construct validity

**Figure 1:**
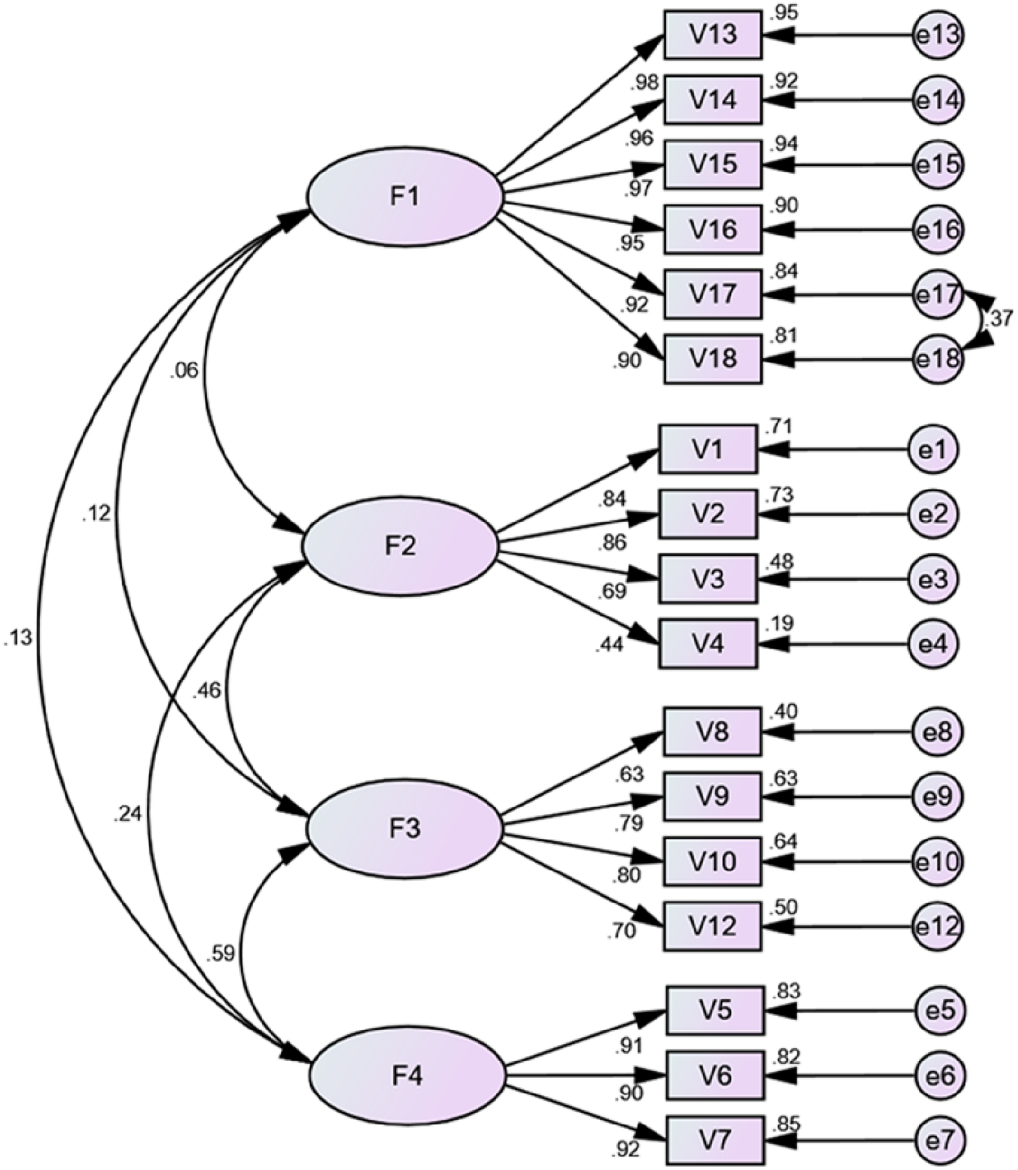
Standardized four-factor structural model of the Chinese version of the SASN (*n* = 288). F1 (professional awareness, six items), F2 (contextual awareness, four items), F3 (personal awareness, four items), F4 (conscientious awareness, three items).

The Bartlett’s test (25) for sphericity of exploratory factor analysis (EFA) was significant (*χ*^2^ = 3883.060, *p* < 0.001) and the KMO index (26) was 0.889. The EFA results indicated that the factor loadings for the items ranged from 0.536 to 0.961 (Table 5). The 4-factor distribution was based on EFA, the CFA model was constructed using AMOS, and the model fit was analyzed. According to the Modification Index (MI), there was one modification to the original model: e17 and e18. Fit indices of the four-factor model (*χ*^2^/df = 2.264, GFI =0.905, CFI = 0.970, TLI = 0.964, RMSEA = 0.066, SRMR = 0.040). The results indicated that the fit of the 4-factor model was statistically acceptable. The regression coefficients are shown in Figure 1.

**Table 5:** Factor loadings of the Chinese version of the SASN (items = 17, *n* = 261).

Item	F1	F2	F3	F4
V1		0.901		
V2		0.860		
V3		0.823		
V4		0.536		
V5				0.890
V6				0.869
V7				0.861
V8			0.750	
V9			0.741	
V10			0.811	
V12			0.664	
V13	0.946			
V14	0.931			
V15	0.961			
V16	0.937			
V17	0.938			
V18	0.910			
Eigenvalue	6.279	3.987	2.007	1.005
Explaining the variance	36.936	23.455	11.804	5.912

### 4.5 Reliability analysis

The SASN overall Cronbach alpha coefficient was 0.873 (95% CI:0.857–0.888), and its four-factor Cronbach coefficients are 0.979, 0.808, 0.820, and 0.918, respectively. Two weeks later, the 30 nurses who participated in the first survey rejoined the survey, and the Spearman correlation coefficient between the retest scores was 0.834 (*p* < 0.001).

### 4.6 Analysis of influencing factors

The results of multiple regression analyses showed statistically significant correlations between overall scores on the nurses’ self-awareness scale and gender, age, work experience, and job title (Table 6). The score of the “professional awareness” subscale was related to gender, age, working experience, job title, and psychological distress. While the score of the “contextual awareness” subscale was related to age, average monthly income, working experience, and psychological distress. Gender, age, working experience, and job title were all associated with scores on the “personal awareness” and “consciousness awareness” subscales.

**Table 6:** Effect of sociodemographic factors on SASN total and subscale scores: multiple regression analysis (*N* = 408).

Model	*B*	SD	Beta	*t*-test (df)	*p*-value
Total score					
Constant	71.074	5.732	—	12.399 (7)	<0.001
Gender	5.938	2.077	0.136	2.859 (7)	0.004
Age (years)	−0.654	0.124	−0.351	−5.271 (7)	<0.001
Education level	−0.940	1.308	−0.036	−0.718 (7)	0.473
Average monthly income (yuan)	1.400	0.887	0.078	1.579 (7)	0.115
Working experience (years)	7.458	0.933	0.524	7.991 (7)	<0.001
Job title	−2.147	1.013	−0.136	−2.119 (7)	0.035
Psychological distress	−0.010	0.053	−0.009	−0.193 (7)	0.847
Professional awareness					
Constant	25.452	2.186	—	11.644 (7)	<0.001
Gender	3.095	0.792	0.181	3.908 (7)	<0.001
Age (years)	−0.196	0.047	−0.269	−4.145 (7)	<0.001
Education level	−0.595	0.499	−0.058	−1.194 (7)	0.233
Average monthly income (yuan)	0.185	0.338	0.026	0.548 (7)	0.584
Working experience (years)	3.096	0.356	0.557	8.700 (7)	<0.001
Job title	−1.008	0.386	−0.163	−2.610 (7)	0.009
Psychological distress	−0.048	0.020	−0.107	−2.364 (7)	0.019
Contextual awareness					
Constant	18.134	2.718	—	6.671 (7)	<0.001
Gender	−0.998	0.985	−0.051	−1.013 (7)	0.312
Age (years)	−0.274	0.059	−0.330	−4.665 (7)	<0.001
Education level	−0.068	0.620	−0.006	−0.109 (7)	0.913
Average monthly income (yuan)	0.934	0.421	0.116	2.220 (7)	0.027
Working experience (years)	1.551	0.443	0.244	3.505 (7)	0.001
Job title	−0.182	0.480	−0.026	−0.379 (7)	0.705
Psychological distress	0.060	0.025	0.118	2.381 (7)	0.018
Personal awareness					
Constant	15.820	1.468	—	10.774 (7)	<0.001
Gender	1.908	0.532	0.171	3.586 (7)	<0.001
Age (years)	−0.115	0.032	−0.241	−3.614 (7)	<0.001
Education level	−0.326	0.335	−0.049	−0.974 (7)	0.330
Average monthly income (yuan)	0.177	0.227	0.039	0.781 (7)	0.435
Working experience (years)	1.834	0.239	0.505	7.672 (7)	<0.001
Job title	−0.548	0.259	−0.136	−2.111 (7)	0.035
Psychological distress	−0.007	0.014	−0.026	−0.547 (7)	0.585
Conscientious awareness					
Constant	11.668	0.900	—	12.97 (7)	<0.001
Gender	1.933	0.326	0.278	5.93 (7)	<0.001
Age (years)	−0.069	0.019	−0.231	−3.522 (7)	<0.001
Education level	0.050	0.205	0.012	0.242 (7)	0.809
Average monthly income (yuan)	0.103	0.139	0.036	0.742 (7)	0.459
Working experience (years)	0.976	0.146	0.431	6.666 (7)	<0.001
Job title	−0.409	0.159	−0.162	−2.570 (7)	0.011
Psychological distress	−0.015	0.008	−0.082	−1.788 (7)	0.075

In conclusion, the four subscales and the total score were correlated with age and working experience. However, only some subscales of the four subscales were correlated with age, gender, job title, and psychological distress.

## 5 Discussion

### 5.1 Chinese version of the self-awareness scale for nurses

To the best of our knowledge, this study is the first attempt to introduce the scale to measure nurses’ level of self-awareness. After a rigorous cultural adaptation process, we translated the scale into Chinese and validated a scale with sufficient reliability and validity that is particularly suitable for assessing nurses’ self-awareness. Finally, a 17-item Chinese version of the SASN with a four-factor structure was created.

The original scale was a four-factor model containing 18 items. Factor 1 - contextual awareness (including items 1–3), Factor 2 - conscientious awareness (including items 4–7), Factor 3 - personal awareness (including items 8–12), and Factor 4 - professional awareness (including items 13–18). The Chinese version of SASN supports a four-factor model consisting of 17 items. Factor 1-professional awareness (including items 13–18), Factor 2-contextual Awareness (including items 1–4), Factor 3-conscientious awareness (including items 8, 9, 10, 12), and Factor 4-personal awareness (including items 5–7) (Table 5; Figure 1). The number of dimensions is the same as the original scale, but the number of items and the factor attribution of the items are slightly different.

### 5.2 Reasonable explanations for differences from the original scale

First, this study translated and adapted the items according to the Chinese expressions, which may have affected the structure of the original scale to some extent. Second, self-awareness belongs not only to psychology, but it belongs to a multidisciplinary field of study. For example, in the field of cognition, a high degree of self-awareness may also protect attentional resources and counteract the deleterious effects of chronic stress on working memory (31). Furthermore, data from neuroimaging studies suggest that inducing self-awareness activates prefrontal cortex regions and helps regulate attention in that region related to working memory (32). In the field of psychology, an individual’s self-awareness is a psychological expression based on his or her perceptions, emotions, and will about himself or herself and his or her relationship with the external world (33). Overall, self-awareness describes a person’s understanding and perception of their environment (34). For nurses, Rasheed et al. (7) believe that self-awareness is essentially a subjective feeling like reflection and evaluation of one’s nursing work. Therefore, the understanding of self-awareness may vary across domains and cultural contexts. Meanwhile, the results of this study showed that Cronbach’s alpha coefficient increased significantly after the removal of item 11 (Table 3), and the content validity of the Chinese version of the SASN was assessed by experts, the I-CVI of item 11 was 0.571. Therefore, the expert group decided to remove item 11.

In addition, item 4 also changed the original dimension, probably because items 1–4 were patient-centered. Clinical care was patient-centered, so patient-centered situational awareness was essential. Finally, the nurses in our study had a high level of self-awareness. The possible reason for this is that a large percentage of nurses in this study had more than 11 years of professional experience (45.9%) and had undergraduate education (78.5%) (Supplementary materials). Similar studies have pointed out that the more experienced and educated nurses are the more significant the positive effect on their self-awareness (12, 19).

### 5.3 Explanation of the relevance of the four dimensions to self-awareness

Self-awareness is an important tool to measure the professional development of nurses [7]. Professional awareness is described as being related to the recognition of one’s profession and the response to different nursing challenges (19). Nurses now assume many roles, which require them to continuously improve their professional knowledge and skills to ensure the safety of patients (35). As the largest group of nursing staff, nurses undertake important medical tasks such as patient assessment and 24-h nursing care (36). To a large extent, this requires nurses to have certain professional skills and expertise, such as being able to judge a patient’s condition promptly based on vital signs, thereby promoting better care. To a certain extent, this reflects the professional awareness of nurses. Based on the available literature and results, we still named factor 1 “professional awareness” and explained 36.936% of the total variance, indicating that it was the most important part of nurses’ self-awareness.

Contextual awareness involves an awareness of the personal and interpersonal factors that influence a situation (19). Meanwhile, results from a mixed study of nursing students suggest that being a good nurse is related to relationships and communication (37). Factor 2 has a patient-centered component, so we still name it “contextual awareness.” In Rasheed’s et al. (12) qualitative study, nurses considered contextualization to be part of self-awareness. Thomson et al. (36) interviewed six psychiatric nurses and concluded that situational awareness has an important role in contextualized care. If nurses are not aware of the underlying factors that affect them personally, interpersonally, this may hinder their ability to provide truly effective patient-centered care (38). Clinical nurses’ workloads, work time constraints, and personal stressors may affect their ability to understand contextualization. For this reason, future research may place more emphasis on this aspect of contextualization (9, 12, 36, 38).

Self-awareness is also a tool to measure nurses’ personal development and is described as the process of examining an individual’s thoughts, feelings, and emotions, all of which can affect their overall health and relationships with others (12). Stovall (39) suggests that everyone should emphasize the role of professional self-awareness by understanding their role, and their career. Self-awareness is essential for personal and professional development and for the development of a genuine nurse–patient relationship (10, 40). It is important to experience self-awareness and then use it healthily. It is the first step to taking better care of yourself and helping you to be resilient (7). So the personal awareness was within the scope of self-awareness and was closely related to it.

As clinical workers, nurses need to have a sense of responsibility. A sense of responsibility helps them to know their role and responsibilities in a given situation and to be able to act accordingly in their care (8). Self-awareness requires an awareness of responsibilities as an individual, each of which requires a different potential to manage the situation (12). Therefore, factor 4 was still named “conscientiousness awareness.”

### 5.4 Analysis of factors influencing self-awareness for nurses

In this study, nurses’ self-awareness was related to gender (Table 6). According to a related article (41), gender differences in self-awareness are primarily brought on by differences in people’s upbringing, social roles, and other factors due to cultural influences, rather than by biological differences between men and women. In traditional Chinese culture, women are frequently advised to be kind, avoid disagreement, demand cooperation, and maintain modesty when being socialized. The intention is for women to become more self-aware than men through maintaining healthy relationships, improving their capacity to comprehend others’ emotions during the socialization process, and valuing self-expression in public. In this culture, women also need to exercise more restraint, stillness, and reflection (41).

This study is consistent with previous findings (9, 19) that nurses’ self-awareness is affected by age, which may be since older clinical nurses tend to have more work and life experience, and greater self-awareness. Furthermore, this study found that self-awareness can have an impact on mental health. This may be attributed to the fact that self-awareness itself can help to understand and change one’s mental state (42–44).

### 5.5 Limitations

Limitations that should be considered in this study include: (1) The study participants were part of the Northeast China nurses. Due to the differences between the north and south of China, these results are not fully representative of all nurses in China. (2) Measures of self-awareness can only reflect a subjective judgment of the participant at the time, so the results of the study should be interpreted with caution.

## 6 Conclusion

In summary, the Chinese version of the SASN consists of 17 items supporting a four-factor structure with good reliability and validity, and it can be utilized to measure the level of self-awareness among Chinese nurses. These findings were important because this was the first study to create a scale to assess the level of self-awareness among Chinese nurses. In this study, nurses’ self-awareness was related to gender, age, and work experience. In future studies, the scale should be applied to different regions to explore the relevant factors affecting nurses’ self-awareness and provide a theoretical basis for further research.

## Data availability statement

The raw data supporting the conclusions of this article will be made available by the authors, without undue reservation.

## Ethics statement

The studies involving humans were approved by the Ethics Committee of Jinzhou Medical University (approval number: JZMULL2021009). The studies were conducted in accordance with the local legislation and institutional requirements. The participants provided their written informed consent to participate in this study.

## Author contributions

QC: Conceptualization, Data curation, Formal analysis, Methodology, Validation, Writing – original draft, Writing – review & editing. CL: Conceptualization, Data curation, Funding acquisition, Methodology, Supervision, Writing – review & editing. JL: Data curation, Validation, Writing – review & editing. ZJ: Funding acquisition, Supervision, Writing – review & editing.
