# Supplementary material for: Translation and validation of the Chinese version of the Self-awareness Scale for Nurses
Source: Front Public Health. 2024 Apr 17;12:1352983. doi: 10.3389/fpubh.2024.1352983 (PMC11061370; doi:10.3389/fpubh.2024.1352983)
Supplement: Supplementary file 1 [file Table_1.DOCX]

**Table 1** Demographic information (N = 549).

| **Variables** | **n** | **%** |
| --- | --- | --- |
| **Age** (mean, standard deviation) | 34.36±7.35 | |
| **Gender** |  |  |
| Male | 8 | 1.5 |
| Female | 541 | 98.5 |
| **Educational level** |  |  |
| Technical secondary school | 10 | 1.8 |
| Junior college | 100 | 18.2 |
| Undergraduate | 431 | 78.5 |
| Postgraduate and above | 8 | 1.5 |
| **Working experience(yea**r) |  |  |
| ≤1 | 43 | 7.8 |
| 2-5 | 91 | 16.6 |
| 6-10 | 163 | 29.7 |
| ≥11 | 252 | 45.9 |
| **Job title** |  |  |
| Nurse | 105 | 19.1 |
| Junior Nurse Practitioner | 227 | 41.3 |
| Supervising Nurse Practitioner | 164 | 29.9 |
| Deputy Chief Nursing Officer and above | 53 | 9.7 |
